# Clinical and Experimental Dental Research celebrates 5 years and the relay baton can be handed over

**DOI:** 10.1002/cre2.271

**Published:** 2019-12-27

**Authors:** Asbjørn Jokstad

In the spring of 2015, the inaugural issue of *Clinical and Experimental Dental Research* included a somewhat optimistic vision that we hoped to count the journal as a prestigious scientific journal in dentistry and orofacial medicine within the next 5 years (Jokstad, 2015).

**Figure 1:**
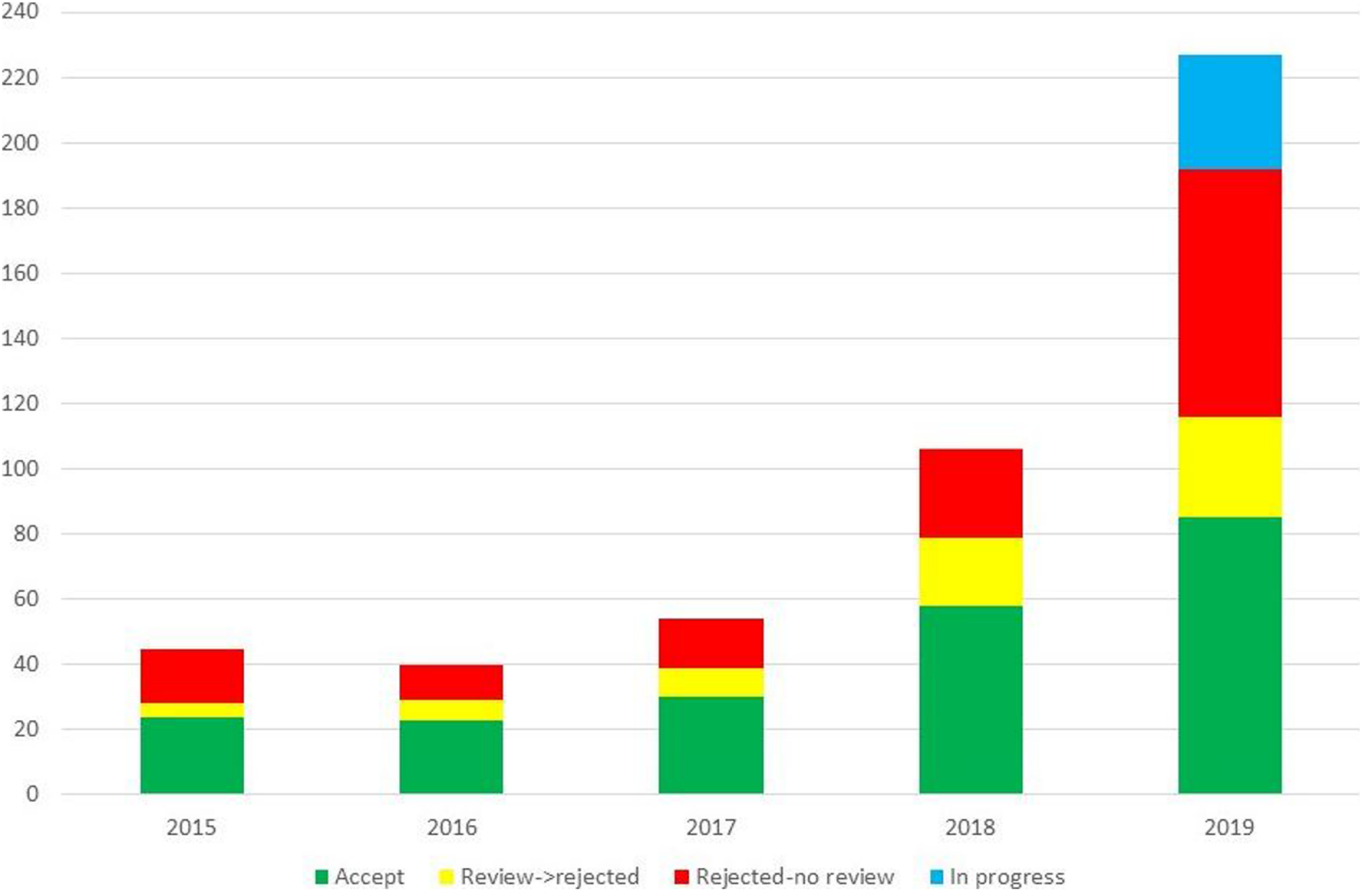
The number of manuscript submissions received by *Clinical and Experimental Dental Research* over the first 5 years (*n* = 510). Accepted articles (*n* = 225), peer‐reviewed and rejected (*n* = 69), immediate rejection without a peer‐review (*n* = 14 7), peer‐reviewed and contact lost with authors (*n* = 34), peer‐review in progress (*n* = 35)

Although today I would have chosen "trustworthy" rather than "prestigious," subtle signs are suggesting that we have reached this goal. The number of manuscript submissions that we received in 2018 doubled from the approximately fifty submissions received annually in the three previous years. It doubled again in 2019 (Figure 1). The journal is ranked as # 34 out of 167 dental journals in terms of verified peer‐reviews over the last twelve months, according to Publons (https://publons.com/journal/?asjc=150). Finally, two influential scientometric indices provide satisfactory marks for the journal, that is, SCImago (https://www.scimagojr.com/journalsearch.php?q=21100843003&tip=sid) and Elsevier/Scopus (Citescore, SNIP, and SJR) (https://www.scopus.com/sourceid/21100843003).

The journal enters now a new phase with a high volume of manuscript submissions, and there is a need for more than just one full‐time professor in academia who, in spare time, attempts to manage a stream of submissions on a 24/7 schedule. It is not so much an issue to browse through new submissions for triaging, but more to set aside time to provide a considerate and polite explanation and perhaps even advises for manuscript improvement to disappointed authors in case of a rejection. The same practice applies to manuscripts that have been peer‐reviewed with a recommendation to reject, although in this circumstance, the authors receive a more generic letter containing something along “in light of the negative criticism from the referees and associate editor detailed below, I am sorry to decline further considerations, etc."

However, the most considerable administrative burden and significant challenge for an editor today are to identify the altruistic assistance from skilled and dedicated peer‐reviewers. Our peer‐review process was sometimes delayed because of a lack of response to invitations sent to potential referees. We experienced even referees who had initially agreed to peer‐review, but next failed to provide the feedback to the journal. Yet, as a member of the Committee on Publication Ethics (COPE), we adhered to their guidelines for ethical publication and tried our best to find a suitable peer‐reviewer (COPE, 2019).

The challenge is common in many other scientific journals, and it applies both for undertaking peer‐reviews and for providing quality peer‐reviews (Breuning, Backstrom, Brannon, Gross, & Widmeier, 2015; Willis, 2016). Agreed, that recognition in Publons may have helped to attract more potential peer‐reviewers. Still, I have failed to observe on a group basis that peer‐reviewers who tick off the box to claim recognition in Publons provide more constructive and comprehensive peer‐reviews versus those who do not. I have also failed to find any literature that has explored this issue. An open peer‐review process is, of course, feasible either in the pre‐publication or the post‐publication stage. Still, some journals have had mixed experiences, and some troubling stories about how perceived defamatory statements have spiraled to court actions have been detailed on http://www.retractionwatch.com. I judge that open peer‐reviewing may perhaps be sustainable in academic societies with limited stakeholders, but risk becoming twisted out of all proportions if available publicly. I do, however, concur with the statement that it is high time that efforts and resources are allocated to promote scientific integrity via journal peer‐review data (Lee & Moher, 2017).

Nobody has found the magic formula for how to revive the tradition‐honored important role of peer‐reviewing. There are some indications that scientific journals that are organized and managed by learned societies rather than by commercial publishers have fewer problems. One narrative emphasizes that peer‐reviewing was initially a social practice within scholarly communities committed to mission‐driven publishing (Moxham & Fyfe, 2018). A further claim is that the only sustainable solution, in the end, is that the scholarly communities must resume the responsibilities for scientific publishing, which the commercial enterprises absorbed during the postwar era (Fyfe et al., 2017).

A perceived threat with OA is the unfortunate industry labeled predatory publishing (Jokstad, 2017). Regrettable, predatory behaviors are not limited only to publishers. We experienced withdrawals of more than thirty submissions that went through a full peer‐review process with recommendations for minor or major corrections and an invitation for resubmission. Some authors explained (sudden) lack of funding, while others simply stopped all further communication, including responding to inquiries about a formal withdrawal of the submitted manuscript. Interestingly, several of these articles have since appeared more or less unchanged on www. Although COPE recommends that we may choose to express our concerns to the authors' institutions, we never initiated such actions. Yet, one cannot stop to wonder about the integrity of the research considering their demonstrated disregard of publication ethics. One author group submitted a manuscript and following three rounds of revisions, the authors requested a withdrawal of the manuscript, which was granted to them without any questions. This manuscript that was peer‐reviewed by our reviewers can be found today as a preprint full text version with a doi on bioRxiv. According to the file info/history tab the article was uploaded a week before the withdrawal request was sent to CEDR. Moreover, the title appears also on the website of an OA journal (ISSN: 2328‐5249). However, it is likely a great disappointment for the authors that this journal closed before their article was published as a fully indexable article. Another story, which calls for more sympathy, was colleagues in Japan who submitted to us an article that was already on the website of a predatory publisher. The author team was shocked when they received a notification of acceptance within 24 hours and became suspicious. Following some correspondence with “the publisher” they received instructions to transfer a lump sum to a bank account to make the article "disappear". Not unsurprisingly, the payment defaulted so the research team remains stuck with the online article. Unfortunately, any serious publisher will decline a republication of their work due to fears of allegations of copyright infringement. The key message in these narratives is that, currently, it is more important than ever to choose the right journal for publishing research, and there are excellent resources on www to assist you in this respect (https://thinkchecksubmit.org).

The efforts of managing the approximately 500 submissions received over these first 5 years have been facilitated by a phenomenal team of associate editors and a huge thank you is deserved (https://onlinelibrary.wiley.com/page/journal/20574347/homepage/editorialboard.html). A heartfelt thank you also goes to all colleagues who have responded to invitations to peer‐review for the journal. Your contributions to quality‐assure the published articles have been indispensable. Finally, thank you to all authors who choose Clinical and Experimental Dental Research as the vehicle for presenting your research. It has been an honor and a privilege to serve you.
